# Association and Functional Analyses Revealed That PPP1R3B Plays an Important Role in the Regulation of Glycogen Content in the Pacific Oyster *Crassostrea gigas*

**DOI:** 10.3389/fgene.2019.00106

**Published:** 2019-02-14

**Authors:** Sheng Liu, Li Li, Jie Meng, Kai Song, Baoyu Huang, Wei Wang, Guofan Zhang

**Affiliations:** ^1^Key Laboratory of Experimental Marine Biology, Institute of Oceanology, Chinese Academy of Sciences, Qingdao, China; ^2^Laboratory for Marine Biology and Biotechnology, Qingdao National Laboratory for Marine Science and Technology, Qingdao, China; ^3^University of Chinese Academy of Sciences, Beijing, China; ^4^Laboratory for Marine Fisheries Science and Food Production Processes, Qingdao National Laboratory for Marine Science and Technology, Qingdao, China; ^5^National and Local Joint Engineering Laboratory of Ecological Mariculture, Qingdao, China; ^6^Center for Ocean Mega-Science, Chinese Academy of Sciences, Qingdao, China

**Keywords:** oyster, glycogen content, protein phosphatase 1 regulatory subunit 3B (PPP1R3B), gene function analyses, associated SNPs

## Abstract

The Pacific oyster (*Crassostrea gigas*) is one of the most important aquaculture species worldwide. Glycogen contributes greatly to the special taste and creamy white color of oysters. Previous genome-wide association studies (GWAS) identified several single nucleotide polymorphism (SNP) sites that were strongly related to glycogen content. Genes within 100 kb upstream and downstream of the associated SNPs were screened. One gene annotated as protein phosphatase 1 regulatory subunit 3B (PPP1R3B), which can promote glycogen synthesis together with protein phosphatase 1 catalytic subunit (PPP1C) in mammals, was selected as a candidate gene in this study. First, full-length CgPPP1R3B was cloned and its function was characterized. The gene expression profiles of CgPPP1R3B in different tissues and seasons showed a close relationship to glycogen content. RNA interference (RNAi) experiments of this gene *in vivo* showed that decreased CgPPP1R3B levels resulted in lower glycogen contents in the experimental group than in the control group. Co-immunoprecipitation (Co-IP) and yeast two-hybrid (Y2H) assays indicated that CgPPP1R3B can interact with CgPPP1C, glycogen synthase (CgGS) and glycogen phosphorylase (CgGP), thus participating in glycogen metabolism. Co-sedimentation analysis *in vitro* demonstrated that the CgPPP1R3B protein can bind to glycogen molecules directly, and these results indicated the conserved function of the CgPPP1R3B protein compared to that of mammals. In addition, thirteen SNPs were precisely mapped in this gene. Ten of the thirteen SNPs were confirmed to be significantly (*p* < 0.05) related to glycogen content in an independent wild population (*n* = 288). The CgPPP1R3B levels in oysters with high glycogen content were significantly higher than those of oysters with low glycogen content, and gene expression levels were significantly associated with various genotypes of four associated SNPs (*p* < 0.05). The data indicated that the associated SNPs may control glycogen content by regulating CgPPP1R3B expression. These results suggest that CgPPP1R3B is an important gene for glycogen metabolic regulation and that the associated SNPs of this gene are potential markers for oyster molecular breeding for increased glycogen content.

## Introduction

The Pacific oyster (*Crassostrea gigas*) is one of the most vital aquaculture species worldwide. Glycogen, a stored form of glucose, is a very important quality trait for oysters because it is responsible for the creamy white color and special taste of the oyster. In addition, glycogen is related to reproductive success and stress tolerance (Whyte and Englar, 1982; Li L. et al., 2018). The heritability of glycogen content in oyster was 0.29 ± 0.02, indicating that it is genetically controlled (Liu et al., 2019), meanwhile, glycogen content is a quantitative trait with high variation among individuals and may affected by many genes and pathways. Hence, genetic improvement of glycogen content is necessary and feasible. Glycogen content is a carcass trait that cannot be measured without killing the animal. Indirect selective breeding for glycogen content by morphological traits seems impossible because of the weak correlation between these characteristics, as shown in several studies (Li et al., 2017; Li C. et al., 2018). Thus, molecular breeding is indispensable for the genetic improvement of glycogen content, which requires elucidation of the underlying genetic basis and the identification of genetic variations.

Glycogen metabolism pathway and physiology in oysters were studied a lot. Berthelin et al. (2000a,b) studied glycogen storage cell isolation, distribution and physiological properties. Several key genes in the glycogen metabolic pathway, such as glycogen synthase (GS), glycogen phosphorylase (GP) (Bacca et al., 2005), phosphoglucomutase (PGM) (Tanguy et al., 2006), glycogenin (Li et al., 2017) and the regulatory gene GSK3β (Zeng et al., 2013), were cloned. Glycogen content showed seasonal fluctuations along with gonad development, the gonads and labial palp had relatively high glycogen levels (Berthelin et al., 2001). Glycogen-related genes show expression variations in different seasons as well as tissues. Furthermore, elucidation of genetic variations of glycogen metabolic genes and their relationships with glycogen content is needed for molecular breeding. Association studies, including genome-wide association studies (GWAS) and candidate gene association analysis, are widely utilized for complex trait dissection and marker screening in crops and livestocks (Li et al., 2013). Although numerous and high frequencies of single nucleotide polymorphisms (SNPs) in the oyster genome were identified by transcriptome and whole genome re-sequencing (Wang J.F. et al., 2015; Li L. et al., 2018), few have been reported on glycogen metabolic genes associated SNPs in oysters. Candidate gene association studies of oyster identified several SNPs in or near GS, GP and glycogen debranching enzyme (GDE) that were significantly associated with oyster glycogen content (She et al., 2015; Liu et al., 2017).

Genome-wide association studies could systematically uncover markers and genes related to target traits, which is powerful and informative and will be beneficial for molecular breeding (McCarthy et al., 2008; Manning et al., 2012; Li et al., 2013; Gutierrez et al., 2018a). GWAS for growth, sex determination, shell color and disease resistance had been conducted in several aquatic animals, such as carp (Zhou et al., 2018), Atlantic Salmon (Correa et al., 2017; Covelo-Soto et al., 2017), Rainbow Trout (Gonzalez-Pena et al., 2016),Yesso Scallop (Zhao et al., 2017) as well as Pacific oyster (Gutierrez et al., 2018a,b). Previous GWAS for nutritional quality traits using 427 oysters were conducted by our research group (Meng et al., unpublished), which revealed three genomic regions, including more than 100 SNPs related to glycogen content. Genes within 100 kb upstream and downstream of the associated SNPs were screened (Supplementary Table S1). One gene annotated as protein phosphatase 1 regulatory subunit 3B (PPP1R3B), with six associated SNPs in or near the gene, was selected as a candidate gene related to glycogen content for further study based on the known function in mammals. PPP1R3B, a glycogen-targeting subunit, forms a holoenzyme together with PPP1C and is believed to be related to the dephosphorylation regulating GS and GP in mammals (Newgard et al., 2000; Stender et al., 2018). Thus, GS is converted from an inactive form to an active form, while GP is converted from an active form to an inactive form. Consequently, increased glycogen accumulation and inhibited glycogen breakdown are observed. To date, the function of invertebrate PPP1R3B remains unclear. Thus, CgPPP1R3B function and genetic variations need to be elucidated.

In this study, we integrated forward and reverse genetic methods to help elucidate the function of this gene and its value in molecular breeding for glycogen content. We precisely mapped the glycogen-associated SNPs in CgPPP1R3B by cloning and validated these SNPs in an independent population. RNA interference (RNAi), overexpression of target genes, and protein interactions with known glycogen metabolic genes were assessed to observe the corresponding changes in glycogen content (reverse genetic methods). We detected gene expression among different genotypes of SNPs and among individuals with different glycogen contents (forward genetic method). We focused on functional studies of the CgPPP1R3B gene in regulating glycogen metabolism as well as genetic variations in or near CgPPP1R3B and their potential for improving oyster glycogen content. This study not only uncovered the genetic basis underlying glycogen content but also developed markers that could be used in molecular breeding for glycogen content in the Pacific oysters.

## Materials and Methods

### Ethics Statement

The Pacific oyster is broadly distributed in the intertidal or sub-tidal areas of the marine environment. The oysters used in this study were collected from wild populations or cultured population derived from artificial breeding by the authors. This study was approved by the Animal Care and Use Committee of Institute of Oceanology, Chinese Academy of Sciences.

### Animals, Sampling and Glycogen Measurement

Oysters used in this study were cultured in the lantern net which was suspended on a maritime longline rope and oysters were taken from the sea to the lab before sampling. There are four parts of oysters used in his study. The first part oysters were sampled from an 16 months old cultured population (shell height = 80.4 ± 1.7 mm) in October that was used to determine the gene expression profiles of different tissues, including gonad, labial palp, gill, mantle, visceral mass and adductor muscle. The second part oysters were used for analysis of the gene expression profiles in different seasons, gonads of 15 adult cultured oysters (18–27 months old, shell height = 84.4–100 mm) were sampled in January, April, July and October, respectively. The third part oysters were used for the RNAi experiments, 6-month old cultured oysters (shell height = 71.0 ± 1.4 mm) were used. The fourth part oysters were used for association analysis, a wild population (*n* = 288) of spat oysters was caught in July, separated into single individuals and cultured in lantern nets with the same density to eliminate possible environmental effects. Oysters which reached a commercial size (18 months old, shell height = 87.4 ± 0.8 mm) were sampled in next February (adductor muscle for subsequent DNA extraction, the left flesh for RNA extraction and glycogen measurement), when glycogen content is relatively high and stable. Gonad development stage were determined based on experience and seawater temperature record according to (Lango-Reynoso et al., 2006), oysters used in each experiment were in the same gonad developmental stage.

For glycogen content measurement of abovementioned oysters, corresponding tissues or the flesh of the oysters was homogenized with liquid nitrogen to powder by a mortar and then freeze dried for 48 h. Approximately 0.1 g of dried flesh powder was used and glycogen measurement was determined by near-infrared reflectance spectroscopy, which is high throughput and more accurate than the traditional method (Wang W.J. et al., 2015).

### Gene Cloning and Bioinformatics Analysis

Full-length CgPPP1R3B and CgPP1C were cloned by rapid amplification of cDNA ends (RACE). All the primers used in this study were listed in Supplementary Table S2. Open Reading Frame Finder^1^ was used to analyze coding sequences and the corresponding deduced polypeptides they encoded. The UniProt database was used to predict protein domains^2^. Protein sequences from different species were downloaded from NCBI^3^. A phylogenetic tree was constructed with the neighbor-joining algorithm using the program MEGA (Version 6.0). The reliability of the branching was tested using bootstrap resampling (1000 pseudo-replicates). Multiple alignments were completed by DNAMAN (Version 9).

### Gene Expression Profile Detection of Different Tissues and Seasons

CgPPP1R3B expression levels in six tissues (gonad, labial palp, gill, mantle, visceral mass and adductor muscle) in October (*n* = 15 for each tissue) and different seasons (*n* = 15) for gonads were determined by real-time PCR (RT-PCR). Total RNA was isolated using an RNAprep Kit (Tiangen, Beijing) according to the manufacturer’s instructions. The RNA integrity and concentration were tested by 1% agarose gel electrophoresis and NanoDrop 2000 spectrophotometry, respectively. cDNA was synthesized using a Prime Script RT Kit (TaKaRa, Dalian). RT-PCR was performed on a 7500 Fast Real-Time PCR System (ABI, United States) using a SYBR Green Master Mix kit (TaKaRa). The primers used for the RT-PCR analysis are listed in Supplementary Table S2. The elongation factor (EF) gene was chosen as an internal control, and each result represents the mean of three replicates.

### Plasmid Construction, Cell Culture, and Transfection

For the generation of tagged protein for further functional studies, the open reading frame (ORF) regions of CgPPP1R3B, CgPPP1C, CgGS, and CgGP were amplified using Phusion High-Fidelity DNA polymerase (Thermo) with specific primers (Supplementary Table S2). pCMV-Myc (Clontech, United States), pEGFP-N1 (Clontech), and pCMS-EGFP-FLAG plasmids (constructed by our lab) and pET-32a (Biomed, Beijing) were digested with EcoRI, XhoI, XhoI, and EcoRI (New England Biolabs, United States), respectively. The purified PCR products were fused with the purified digested plasmids using the Ligation-Free Cloning System (Applied Biological Materials, Inc., Canada). *Escherichia coli* Trans T1 cells (TransGen, China) were transformed with the fusion mixture and cultured in LB agar plates overnight, and the grown colonies were tested by colony PCR. One clone was confirmed by Sanger sequencing, and the corresponding plasmids were extracted from overnight cultured bacteria using an endo-free plasmid extraction kit (Tiangen).

For co-immunoprecipitation (Co-IP) and the CgPPP1R3B protein overexpression assays, HEK293T cells (ATCC) were cultured in Dulbecco’s modified Eagle’s medium (high glucose) (HyClone). HeLa cells (ATCC, United States) were cultured in modified Roswell Park Memorial Institute (RPMI)-1640 medium (HyClone, United States) for subcellular assays. Both types of media were supplemented with 10% fetal bovine serum (HyClone) and 1× penicillin-streptomycin solution (Solarbio, China). Cells were grown in an atmosphere of 5% CO_2_ at 37°C. Plasmids were transfected into HeLa or HEK293T cells using Lipofectamine 3000 reagent (Life Technologies, United States) according to the manufacturer’s instructions.

### CgPPP1R3B RNAi *in vivo* and Overexpression in HEK293 Cells

For further elucidation of the role of CgPPP1R3B in glycogen metabolism, we performed CgPPP1R3B RNAi experiments. RNAi was conducted by small interfering RNA (siRNA) synthesized by Shanghai GenePharma company. SiRNA sequences are shown in Supplementary Table S2. This experiment was carried out in January, which is thought to be the glycogen accumulation stage (Berthelin et al., 2000b). Oysters were anesthetized by mixed seawater (500 g MgCl_2_ + 5 L seawater + 5 L freshwater) according to Suquet et al. (2009). One hundred and eighty oysters were then randomly divided into three groups of 60 individuals. 100 μl SiRNA (100 ng/μl), neutral control (NC) RNA (100 ng/μl) and H_2_O (blank control, BC) were injected into the adductor muscle; the animals were injected twice on days 0 and 10. After injection, a small amount of gonad and labial palp was sampled for RNA extraction for further gene expression analysis of CgPPP1R3B, CgGS and CgGP by RT-PCR, and the left flesh was sampled for glycogen content measurement. The tissue was sampled for 18 days, and details of sampling are shown in Supplementary Table S3. Overexpression of CgPPP1R3B was conducted in HEK293 cells, which were cultured in 5-cm diameter dish Petri dishes (Corning, United States). CgPPP1R3B-Myc, CgPPP1R3B-FLAG, and CgPPP1R3B-EGFP were transfected, and cells were harvested 24 h later for glycogen measurement by a kit according to the manufacturer’s instructions (Jiancheng, Nanjing). The glycogen content represents the mean of three independent replicates.

### Protein–Protein Interaction Methods

For analysis of the protein interactions of CgPPP1R3B with its catalytic domain CgPPP1C and CgPPP1R3B with CgGS and CgGP, Co-IP and yeast two-hybrid (Y2H) assays were carried out.

#### Co-IP Assays

HEK293T cells were divided between two or more Petri dishes (10 cm diameter, Corning, United States) and cultured for 24 h. Fusion pCMV-Myc/pEGFP-N1 plasmids were co-transfected with vectors expressing FLAG-tagged fusion proteins or empty FLAG vector (control). After 24 h, cells were harvested, and proteins were extracted by cell lysis buffer (Beyotime). Control (input) samples were prepared from the cell lysate, and the remaining lysates were mixed with the anti-FLAG M2 magnetic beads (Sigma, United States) by gently mixing with a roller at 4°C for 1–2 h. The beads were washed twice with PBS and then washed twice with cell lysis buffer. Input and Co-IP samples were mixed with 2× protein SDS-PAGE loading buffer (TaKaRa) and then denatured at 100°C for 5 min. Proteins were analyzed by SDS-PAGE and then by Western blotting using anti-Myc/GFP and anti-FLAG antibodies (Sigma).

#### Y2H Assays

Y2H assays were performed to detect interactions between proteins using the Clontech Matchmaker Gold Yeast Two-Hybrid System (TaKaRa). The fusion plasmids pGADT7 (AD vector) and pGBKT7 (BD vector) were transformed into Y187 and Gold yeast competent cells, respectively. Y187 cells were cultured on selective plates with synthetically defined (SD) medium lacking leucine (SD/-Leu), whereas Gold cells were cultured on plates lacking tryptophan (SD/-Trp). After 48–96 h, yeast were grown on SD/-Leu and SD/-Trp medium and then hybridized in 2× yeast extract peptone dextrose-adenine (YPDA) medium and selected by a double dropout (SD/-Leu/-Trp) plate. Interactions between proteins were detected based on the ability of the hybridized yeast to grow on quadruple dropout (SD/-Ade/-His/-Leu/-Trp) medium supplemented with *X*-α-Gal and aureobasidin A (TaKaRa).

### Glycogen-Protein Co-sedimentation Assay

For analysis of whether the CgPPP1R3B protein could bind to the glycogen molecule, glycogen-protein co-sedimentation assays were performed. Prior to the co-sedimentation assay, recombinant pET-32a/CgPPP1R3B proteins (6x-His Tag) were produced in *E. coli*. Plasmid-ligated pET-32a was transformed into the *E. coli* BL21 (DE3) strain (Vazyme, Nanjing) and cultured on the corresponding LB agar plate. A single bacterial colony was selected and cultured overnight at 37°C in 10 ml LB medium (supplemented with 100 μg/ml ampicillin). In another conical flask with 200 ml LB medium, 4 ml of the abovementioned bacteria was inoculated and cultured to OD600 = 0.6. Protein production was induced by the addition of 1 mM isopropyl β-D-thiogalactoside for 16 h in a 16 °C incubator. Bacterial cells were collected by centrifugation, and the pellets were resuspended in PBS. Then, resuspended bacteria were ultrasonicated and centrifuged for 5 min at 12,000 *g*. The supernatant was used for purification of recombinant protein using a His-tag Protein Purification Kit (Beyotime, Shanghai).

Glycogen-protein co-sedimentation assays were performed as described previously (Kerekes et al., 2014). A total of 400 μl of 0.5 mg/ml recombinant pET-32a/PPP1R3B was incubated with 670 μl of 17.6% (mass/volume) glycogen solution (from oyster, Yuanye) and 1600 μl of buffer for 2 h at 4°C; the buffer contained Tris-HCl (pH 7.5), mercaptoethanol, PMSF, EDTA, and protease inhibitor cocktail (Roche), with concentrations of 10, 1, 1, 1, and 1 mM, respectively. After incubation, 4 ml of extra buffer was added, and the solution was layered over 6.7 ml of 0.25 M sucrose in a 12.5 ml centrifuge tube. After centrifugation, the supernatant and pellet fractions were separated for 90 min at room temperature at 25,700 rpm (100,000 *g*) with a Beckman XL-80K centrifuge. The original pET-32a/vector was used as a negative control experiment and was treated in the same way. After the addition of 2 × SDS buffer, the samples were denatured for 5 min at 100°C and then examined by SDS-PAGE and Western blotting with an anti-His antibody (TransGen, China).

### Subcellular Localization Assay

For detection of the subcellular localization of CgPPP1R3B, CgPPP1C, CgGS, and CgGP, HeLa cells were transfected with CgPPP1R3B-EGFP, CgPPP1C-EGFP, CgGS-EGFP, CgGP-EGFP or pEGFP-N1, and nuclei were counterstained with Hoechst 33342 (blue) (Invitrogen, United States). The membrane was counterstained with Alexa Fluor 594 (red) (Invitrogen, United States). After the nuclei and membrane were stained for 10 min at 37°C, cells were washed twice with PBS and then observed using laser scanning confocal microscopy (Carl Zeiss, Germany).

### Genotyping of Candidate SNPs

Prior to the association analysis, genotyping of the candidate SNPs was performed. Genomic DNA of oysters was extracted from the adductor muscle by sodium laurate as described previously (Cai et al., 2014) with a simple modification. The integrity and concentration of the DNA were tested by 1% agarose gel electrophoresis and NanoDrop 2000 spectrophotometry, respectively. All SNPs used in this study were derived from whole-genome resequencing of 427 individuals (Li L. et al., 2018).

Thirteen SNPs, including six associated SNPs (63644, 63965, 64380, 64742, 67094, and 67096) from the previous GWAS results (Meng, unpublished) and other seven newly identified SNPs (63878, 68341, 64496, 71564, 72487, 77716, and 78604) were genotyped by SNaPshot method with 3730xL DNA Analyzer (Applied Biosystems) in the wild population (*n* = 288).

### Association Studies Among Genotypes, CgPPP1R3B Expression and Glycogen Content

For determination of the association between genotypes of SNPs and glycogen content, a linear regression model was used in SPSS 22.0 software (dominant allele homozygotes assigned 0, heterozygotes assigned 1, and recessive allele homozygotes assigned 2). Analyses of linkage disequilibrium among SNPs were performed according to http://analysis.bio-x.cn/myAnalysis.php (Shi and He, 2005). The association between SNP genotypes and gene expression levels was determined as described above. RNA for association studies were extracted from the same homogenized flesh used for glycogen measurement, and the expression levels of individuals (*n* = 210) with various genotypes were determined by RT-PCR. CgPPP1R3B expression in high-glycogen (*n* = 20) and low-glycogen (*n* = 20) individuals was evaluated to assess their relationships.

### Data Analysis

Glycogen content is shown as the mean ± standard error of the mean (SEM), and gene expression levels were calculated by the 2[-Delta Delta C(T)] method (Livak and Schmittgen, 2001), the data are shown as the mean ± SEM. For RNAi study, one-way ANOVA followed by a *post hoc* multiple comparison (Duncan) was conducted to test the significance of the expression levels or glycogen content among three groups, student’s *t*-test were used in Microsoft Excel for comparing the experiment group with control in CgPPP1R3B overexpression study. In association study, a linear regression model was used in SPSS 22.0 as mentioned above, the *p*-value indicates the significance of the association, *R*^2^ in the model represents the phenotypic variation explained (PVE). Student’s *t*-test were used in Microsoft Excel for comparing the expression level of different genotypes’ individuals or low and high glycogen groups.

## Results

### Gene Functional Studies

#### Gene Cloning, Phylogenetic Tree Construction and Expression Profile

A full-length cDNA of 1717 bp was isolated from oyster cDNA and designated CgPPP1R3B. CgPPP1R3B encodes a 351 amino acid polypeptide (Supplementary Figure S1), with a predicted molecular weight of 39.66 kDa and a predicted carbohydrate binding type-21 (CBM21) domain. Another full-length cDNA was 1171 bp and designated CgPPP1C. CgPPP1C encodes a 328 amino acid polypeptide (Supplementary Figure S2), with a predicted molecular weight of 37.29 kDa, and UniProt blast analysis predicted a PP2A domain (protein phosphatase 2A homologs, catalytic domain).

**FIGURE 1:**
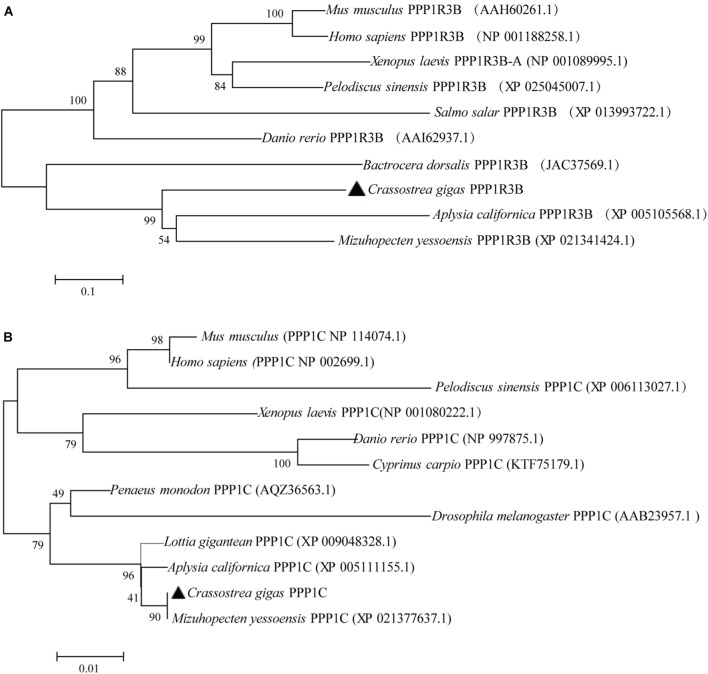
Phylogenetic analysis of the CgPPP1R3B **(A)** and CgPPP1C **(B)** protein of human, mouse, amphibian, reptile, fishes, oyster, and other shellfish, protein sequence were downloaded from NCBI (the serial number after the Latin name of the species). Numbers beside the internal branches indicate bootstrap values based on 1000 replications. The 0.1 and 0.01 scale indicates the genetic distance.

The phylogenetic tree was constructed based on the protein sequences of PPP1R3B and PPP1C from human, mouse, amphibian, reptile, fishes, oyster, and other shellfish (Figure 1). The phylogenetic tree and multiple alignment analysis suggest that CgPPP1R3B and CgPPP1C, should be an ortholog of invertebrate and vertebrate PPP1R3B and PPP1C proteins, respectively. The PPP1R3B sequence varied greatly among species; the similarity of the oyster to mouse protein is 40%, which was relatively low, as shown in Supplementary Figure S3. The CgPPP1C sequence was strongly conserved, with a similarity of more than 90%, as indicated by the multiple alignment analysis shown in Supplementary Figure S4.

CgPPP1R3B expression in different tissues in October showed high levels in the gonad, labial palp and mantle, which have relatively high glycogen contents (Supplementary Figures S5A,B). In different seasons, the expression levels showed similar trends to the glycogen contents. In winter and autumn, the CgPPP1R3B transcript levels and glycogen contents were higher than those in spring and summer (Supplementary Figure S5C).

#### Knockdown of CgPPP1R3B by RNAi Decreased the Glycogen Content

**FIGURE 2:**
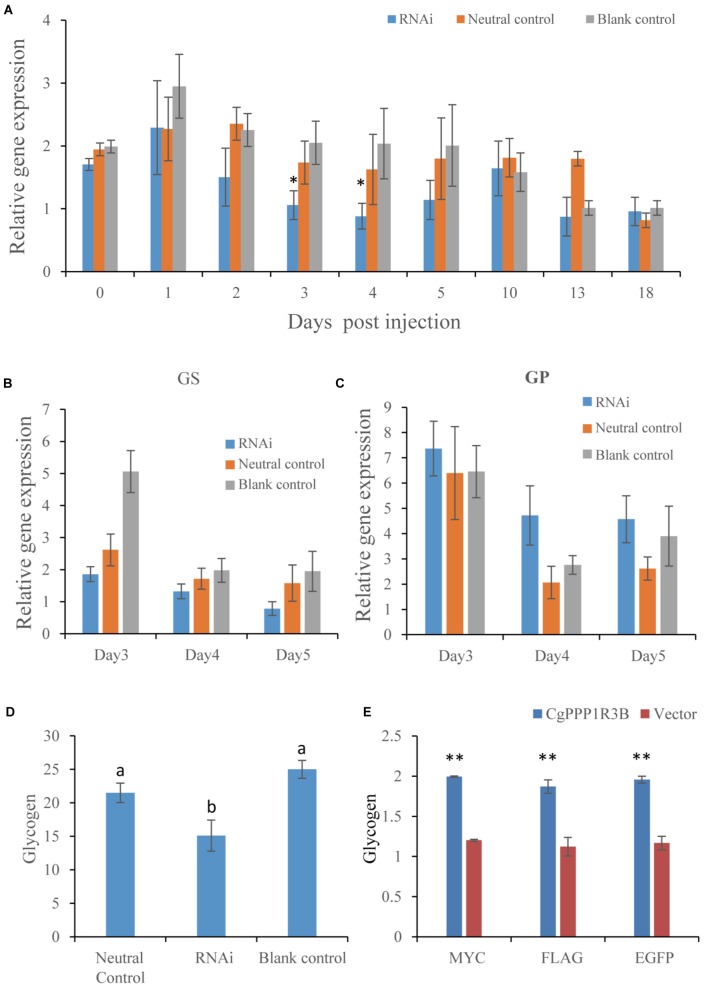
CgPPP1R3B RNAi *in vivo* and overexpression in HEK293T cell. **(A)** CgPPP1R3B expression after RNAi in days post first injection (*n* = 5), oysters were injected twice in day 0 (first) and day 10 (second). The asterisk indicates significant difference (*p* < 0.05) between RNAi and neutral control group. **(B)** CgGS and **(C)** CgGP expression in days 3–5 post first injection (*n* = 5). **(D)** Glycogen content in day 18 post first injection (*n* = 5), different letters of “a” and “b” mean significant difference (*p* < 0.05), glycogen content was percentage of flesh dry weight. **(E)** Fold change of glycogen content in HEK393T cell overexpressed CgPPP1R3B in three kinds of vector (*n* = 3), double asterisk mean glycogen levels of overexpressed group is higher than vector control (*p* < 0.01). Bars in **(A–E)** represent mean ± SEM (standard error of means).

The gene expression level of the RNAi group was lower than that of the NC and BC groups on most days. Knockdown of CgPPP1R3B expression resulted in a strong interference effect 3–4 days post-injection (Figure 2A). These results demonstrated that suppression of CgPPP1R3B slightly decreased CgGS and increased CgGP expression on days 4 and 5 (Figure 2B,C) and decreased glycogen content on the 18th day post-first injection (Figure 2D).

#### Overexpression of PPP1R3B Increased Glycogen Content in HEK293 Cells

CgPPP1R3B was overexpressed in HEK293 cells, and the results showed that glycogen content was significantly higher in the overexpression group than in the control group (vector), and no differences among different vectors were found in improving glycogen content (Figure 2E).

#### CgPPP1R3B Interacted With CgPPP1C, CgGS, CgGP and Glycogen Molecules

**FIGURE 3:**
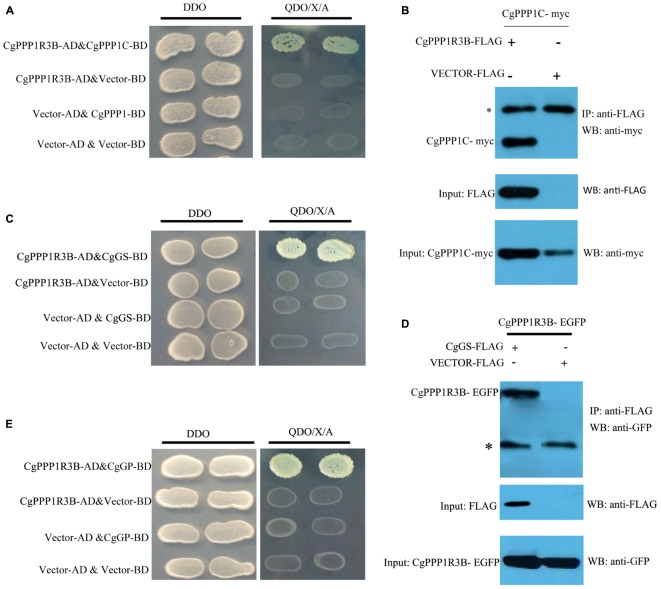
CgPPP1R3B interact with CgPPP1C, CgGS, and CgGP. Interaction between CgPPP1R3B and CgPPP1C were demonstrated by Y2H **(A)** and Co-IP **(B)**. CgPPP1R3B interact with CgGS by Y2H **(C)** and Co-IP **(D)**. CgPPP1R3B interact with CgGP by Y2H **(E)**. DDO: -Leu/-Trp double-dropout media, QDO/X/A: -Ade/-His/-Leu/-Trp quadruple-dropout media with *X*-α-Gal and Aureobasidin A. The asterisk in **(B,D)** indicate the IgG heavy chain.

We conducted Y2H and Co-IP assays to detect the interaction of CgPPP1R3B with its catalytic subunit CgPPP1C (Figure 3A,B). The CgPPP1C binding site was the KRVSF (133–137 aa) motif, as shown by the truncated protein analyses and site-directed (Supplementary Figure S6A) mutagenesis of V135E in CgPPP1R3B, which abrogated this interaction (Supplementary Figure S6B).

**FIGURE 4:**
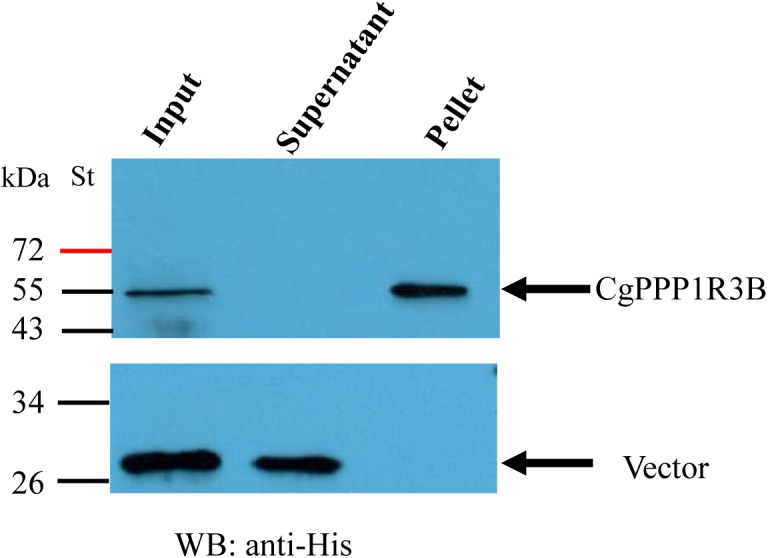
CgPPP1R3B protein bind with oyster glycogen molecule directly *in vitro*. Input refers to sample before centrifugation as a control. After centrifugation, the sediment in the bottom of the tube were named pellet. Input, supernatant, and pellet were tested by western blotting with anti-His. St represents protein molecular mass standards; molecular masses are given in kDa.

**FIGURE 5:**
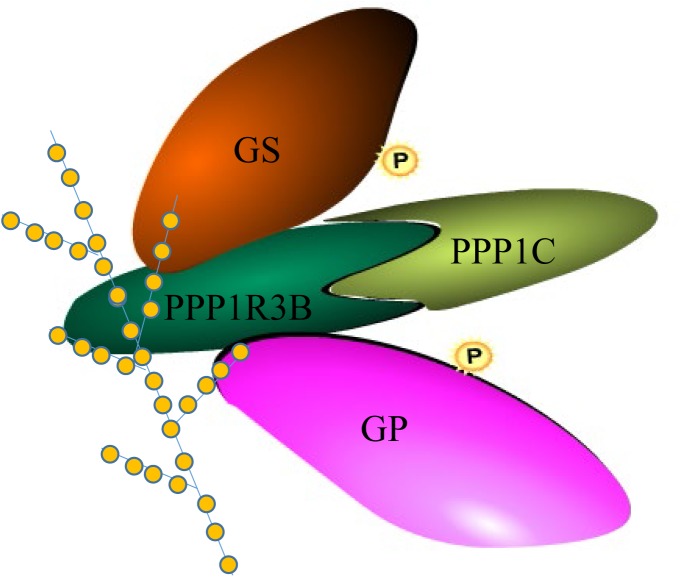
Complex of CgPPP1R3B with CgPPP1C, CgGS, CgGP and glycogen molecule. Four colors of the module represent CgPPP1R3B, CgPPP1C, CgGS, CgGP protein, respectively. “P” in the yellow circle represent possible phosphorylation of the protein. The dendrimer-like molecule represent glycogen with orange circle represent the glucose residue.

Based on a previous study in mammals, we carried out assays to determine whether CgPPP1R3B interacts with key enzymes involved in glycogen metabolism, such as CgGS and CgGP. The results showed that CgPPP1R3B can interact with CgGS in Y2H and Co-IP assays (Figure 3C,D); similar results were obtained for CgGP in Y2H assays (Figure 3E). The interaction domain of CgPPP1R3B with CgGS was not determined because none of the truncated proteins could interact with CgGS (Supplementary Figure S6C). The interaction domain of CgPPP1R3B with CgGP may be the C-terminal of the protein, as indicated by the truncated protein experiments shown in Supplementary Figure S6D. The co-sedimentation of the CgPPP1R3B protein with glycogen molecules showed that they can bind each other *in vitro*, while the pET-32a vector-expressed tag protein did not bind to the glycogen molecules, as shown in Figure 4. Thus, a large complex of CgPPP1R3B, CgPPP1C, CgGS, and CgGP that targets glycogen molecules can form (Figure 5).

#### Subcellular Localization

Finally, to directly assess the subcellular localization of CgPPP1R3B, CgPPP1C, CgGS and CgGP, we performed fluorescence localization analyses. The fluorescent signals representing the CgPPP1R3B fusion proteins were mainly located in the cytoplasm, and CgGS appeared to aggregate together in the cytoplasm. In contrast, CgPPP1C and CgGP were distributed in a dispersed manner in both the cytoplasm and the nucleus (Supplementary Figure S7).

### Association Studies of SNPs in CgPPP1R3B

#### Glycogen Measurement and Genotyping of SNPs in the Wild Population

Glycogen content in the wild population ranged from 14.9 to 49.4% dry weight, with a mean value of 37.5 ± 0.3%, which followed the normal distribution (*p* > 0.05). There was no subpopulation structure of this wild population based on genomic kinship matrix and principal component analysis (unpublished data).

**FIGURE 6:**
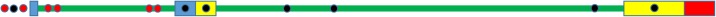
Gene structure of CgPPP1R3B and glycogen content associated SNP location in this gene in the Pacific oyster. From left to right (5′—3′), red dot represent SNPs from GWAS result (from left to right 63644, 63965, 64380, 64742, 67094, and 67096), black ones represent newly identified SNPs (from left to right 63878, 68341, 68496, 71564, 72487, 77716, and 78604). Blue box represent 5′ UTR, yellow box is CDS, red box is 3′ UTR, and green lines are introns.

We mapped the CgPPP1R3B cDNA sequence to the genome, and the relative location of CgPPP1R3B spanned from scaffold 1243–64080 to scaffold 1243–79594. The full-length DNA of this gene was 15515 bp, which is much longer than predicted, with three exons and two introns, as shown in Supplementary Table S4. The precise locations of the 13 SNPs in the genome are presented in Figure 6. Three SNPs were located in the 5′ flanking region (SNP 63644, 63878, and 63965), one in the 5’UTR (SNP 68341), seven in the introns (SNP 64380, 64742, 67094, 67096, 71564, 72487, and 77716) and two in the CDS region (SNP 68496, 78604, synonymous mutation). Six of the SNPs were T/C transitions, four were A/G transitions, two were A/C transversions, and one was a T/G transversion. The minor allele frequency (MAF) was more than 0.05 for most of the SNPs, ranging from 0.09 to 0.47, for SNP 68496 it is 0.02. Genotype and allele frequencies of the 13 SNPs in the wild population are shown in Supplementary Table S5.

#### Association Between SNPs and Glycogen Content

The associations between the genotypes of 13 SNPs and glycogen content are shown in Table 1. SNP 63644 (TT > TC > CC), SNP 63965 (CC > TC > TT), SNP 63878 (TT > TC > CC), SNP 64742 (TT > TC > CC), SNP 67094 (AG > GG > AA), SNP 67096 (AG > GG > AA), SNP 68341 (TT > TC > CC), SNP 71564 (CC > TC > TT), SNP 72487 (AA > AC > CC), and SNP 78604 (TT > TG > GG) were significantly related to glycogen content (*p* < 0.05). These SNPs accounted for 1.4 to 6.3% of the PVE. The minor allele was shown to be the favorable allele of 63644 (T), 63878 (T), 63965 (C), 64742 (T), 68341 (T), 71564 (C), 72487 (A), and 78604 (T). For 67094 and 67096, individuals with favorable alleles had increased glycogen levels, while the heterozygotes had higher glycogen contents than each of the homozygotes. For other significant SNPs, the results showed that favorable allele homozygotes > heterozygotes > unfavorable allele homozygotes.

**Table 1:** Association among genotypes of 13 SNPs and glycogen content in a wild independent population of the Pacific oysters.

SNP ID	Location	Allele	Genotypes	Glycogen (%)	*P*-value	PVE (%)
63644#	5′ flanking	T > C	CC	36.29 ± 0.52	<0.001^∗∗∗^	6.3
			CT	37.73 ± 0.53	
			TT	39.11 ± 0.35	
63965#	5′ flanking	C > T	TT	37.04 ± 0.33	<0.001^∗∗∗^	4.6
			CT	38.08 ± 0.60		
			CC	40.0 ± 0.81		
63878#	5′ flanking	T > C	CC	37.08 ± 0.30	<0.001^∗∗∗^	5.3
			CT	39.55 ± 0.67	
			TT	40.93 ± 1.29	
64380	Intron	A > C	CC	37.28 ± 0.35	0.235	0.5
			AC	38.13 ± 0.70		
			AA	38.69 ± 0.87		
64742#	Intron	T > C	TT	40.41 ± 0.94	0.002^∗∗^	3.5
			CT	38.65 ± 0.70		
			CC	37.16 ± 0.33		
67094#	Intron	G > A	AA	37.21 ± 0.32	0.029^∗^	1.7
			AG	40.20 ± 0.80		
			GG	38.58 ± 1.09		
67096#	Intron	G > A	AA	37.23 ± 0.33	0.043^∗^	1.4
			AG	39.51 ± 0.80		
			GG	38.73 ± 1.02		
68341#	5′ UTR	T > C	CC	36.83 ± 0.39	0.003^∗∗^	3.0
			CT	38.17 ± 0.44		
			TT	40.22 ± 0.97		
68496	CDS(synonymous)	G > A	AG	36.68 ± 1.55	0.62	0.1
			GG	37.43 ± 0.30		
71564	Intron	C > T	CC	38.77 ± 0.68	0.03^∗^	1.6
			CT	37.14 ± 0.4		
			TT	36.91 ± 0.57		
72487	Intron	A > C	AA	39.52 ± 1.06	0.001^∗∗^	3.7
			AC	37.8 ± 0.65		
			CC	36.84 ± 0.36		
77716	Intron	A > G	AG	38.88 ± 0.73	0.46	0.2
			GG	37.23 ± 0.32		
78604#	CDS(synonymous)	T > G	GG	36.00 ± 0.90	0.04^∗^	1.4
			TG	37.37 ± 0.43		
			TT	37.83 ± 0.45		


**In allele column, T > C means T was the favorable allele for glycogen content, genotyping were completed with SNaPshot method. Glycogen content were shown as percentage of flesh dry weight. PVE is the phenotype variation explanation (%). # means that the SNP was also significantly associated with glycogen content in the population for GWAS results (Meng et al., unpublished). Three significance levels were used: ^∗^p < 0.05, ^∗∗^p < 0.01, ^∗∗∗^p < 0.001*.*

#### Relationship Between CgPPP1R3B Expression Levels and Glycogen Content

**FIGURE 7:**
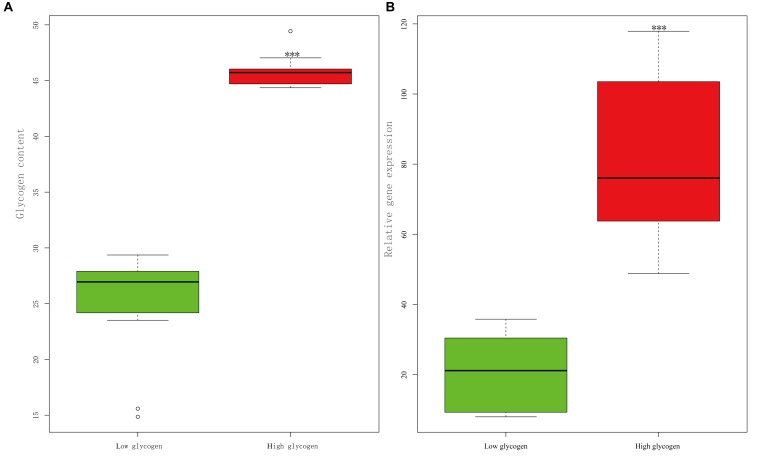
Relationship between relative gene expression level of CgPPP1R3B and glycogen content in the Pacific oyster. **(A)** Glycogen content in high glycogen (*n* = 20) and low glycogen (*n* = 20) group in the wild population. **(B)** Relative gene expression of CgPPP1R3B in high glycogen (*n* = 20) and low glycogen (*n* = 20) group in the wild population. Triple asterisk represent significant difference (*p* < 0.001).

High-glycogen individuals (*n* = 20) and low-glycogen individuals (*n* = 20), whose contents were 45% ± 0.29% and 26% ± 0.96%, respectively, were significantly different (*p* < 0.001). The gene expression of CgPPP1R3B in high-glycogen individuals was significantly higher than that in low-glycogen individuals (*p* < 0.001) (Figure 7).

#### Association Between SNPs and CgPPP1R3B Expression Levels

**FIGURE 8:**
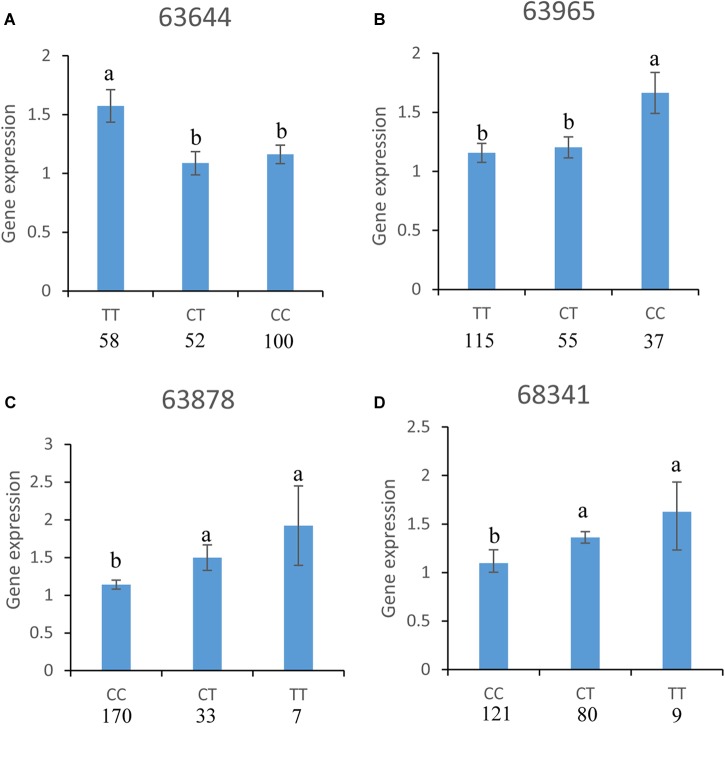
Association between SNPs and CgPPP1R3B expression levels, different genotypes of four SNPs: **(A)** 63644, **(B)** 63965, **(C)** 63878, and **(D)** 68341 were significantly associated with CgPPP1R3B expression levels. Numbers in the bottom of the genotypes were individuals of specific genotypes. The letters “a” and “b” summarize the statistical grouping.

Analyses of the relationships between gene expression levels and 13 SNP loci showed that different genotypes of four SNPs (63644, 63965, 63878, and 68341) were significantly related to CgPPP1R3B expression levels (Figure 8). For SNP 63644, the CgPPP1R3B expression level of favorable allele homozygotes (TT, *n* = 58) was significantly higher than that of heterozygotes (TC, *n* = 52) and unfavorable allele homozygotes (CC, *n* = 100). There was no difference between heterozygotes and unfavorable allele homozygotes. For SNP 63965, different expression levels were observed between favorable allele homozygotes (CC, *n* = 37) and heterozygotes (TC, *n* = 55) or unfavorable allele homozygotes (CC, *n* = 115). There were no significant differences in the transcript levels of heterozygotes and unfavorable allele homozygotes. For SNP 63878, strong evidence of distinct expression levels was found among favorable allele homozygotes (TT, *n* = 7), heterozygotes (TC, *n* = 33) or unfavorable allele homozygotes (CC, *n* = 170) (i.e., TT > CC, TC > CC). For SNP 68341, the difference between the dominant allele homozygotes (TT, *n* = 9) and heterozygotes (TC, *n* = 80) was significant; it was similar between dominant allele homozygotes and recessive allele homozygotes (CC, *n* = 121) (i.e., TT > CC, TC > CC).

#### Effect of Genotypic Combination

The 10 associated SNPs were used to characterize the linkage disequilibrium (LD) pattern. The results showed that the LD block was quite short, and only SNP 67094 and SNP 67096 were located in the same block; other glycogen-associated SNPs were not in the same LD block (Supplementary Figure S8) and showed independent assortment. The genotypic combination of most significantly associated SNPs (63644, 63965, and 63878) showed that the glycogen content was 42.77 ± 0.91, which was 14.1% higher than the average content. Individuals with the most favorable combinations accounted for 4.9% of the total wild population. For individuals with unfavorable combinations, glycogen content was 35.93 ± 0.56, which was 4.2% lower than the average. Not all of the combinations are shown in Table 2 because there are many combinations. Individuals with favorable allele combinations had higher glycogen content than those with a single favorable allele.

**Table 2:** Favorable genotypes combination of three SNPs-63644, 63965, and 63878 in the Pacific oysters.

Combination	Glycogen (%)	Improvement	Count	Percentage
TT+CC+TT/TC	42.77 ± 0.91	14.1%	14	4.90%
CC+TT+CC	35.93 ± 0.56	-4.2%	87	30.40%
Total average	37.5 ± 0.32	0	288	100%


**Improvement means extent of glycogen content of genotype combination higher/lower than the total average. Count means number of individuals with the combination, and the percentage means the proportion of the individuals in the wild population*.*

## Discussion

Based on previous GWAS results, candidate genes and SNPs were acquired. We explored gene functions and validated associated SNPs in an independent wild population. We adapted both forward genetics and reverse genetics methods to further elucidate CgPPP1R3B function in regulating glycogen content and associated variations that may be potentially useful in molecular breeding.

### Identification of PPP1R3B Function

CgPPP1R3B encodes a longer protein than most of its orthologs in invertebrates and vertebrates. Multiple alignment showed that the CgPPP1R3B protein had relatively low similarity (40%) to that of humans (Supplementary Figure S3), whereas for rabbit and mouse, the homologies were 70–85% (Newgard et al., 2000). CgPPP1C encoded a strongly conserved protein with a similar length to that of other animals (Supplementary Figure S4), with more than 90% similarity. The high similarity suggested the conserved function in different taxa of animals. Several glycogen metabolic genes were cloned in the Pacific oyster, and most of the identified glycogen metabolism and corresponding genes show more than 60% similarity to protein sequences of other species, thus indicating a relatively conserved pathway (Bacca et al., 2005; Hamano et al., 2005; Tanguy et al., 2006; Hanquet et al., 2011; Zeng et al., 2013; Li et al., 2017).

For the gene expression profiles, CgPPP1R3B expression increased in tissues with high glycogen contents in October, consistent with the high expression of CgGS observed by Bacca et al. (2005). These results are consistent with the finding that storage cells are mainly distributed in the labial palp, mantle and gonadal area, as shown by periodic acid–Schiff staining and ultrastructural characteristics of isolated storage cells (Berthelin et al., 2000a,b). Glycogen is mainly stored to meet the energy demands of reproduction, and the results also demonstrated that CgPPP1R3B expression and glycogen content were relatively high in the degenerating gametes stage (autumn)and early gametogenesis (winter), decreased accordingly in the growth of gamete stage (spring) and mature and spawning stages (summer). These findings were supported by the corresponding experiments (Berthelin et al., 2001).

The protein interaction results showed that CgPPP1R3B could interact with CgPPP1C, and the interaction motif was proven to be KRVSF (133–137 aa), which is consistent with the binding site of the glycogen targeting subunit (GTS) in mammals (Armstrong et al., 1998). The site-directed mutagenesis of the consensus sequence (V135E, KR**V**SF to KR**E**SF) blocked CgPPP1C binding, which was consistent with the point mutation results for another GTS (Fong et al., 2000). Four types of GTS have been identified in mammals, which share the PP1C and glycogen binding function, with *G*_M_ mainly distributed in muscle, PPP1R3B (*G*_L_) mainly expressed in liver, and PPP1R6 and protein targeting to glycogen (PTG) distributed widely, which reflect tissue type-dependent glycogen metabolism (Printen et al., 1997; Newgard et al., 2000). We screened the oyster genome protein dataset (Zhang et al., 2012) by the KRVSF/KRVVF/RRVSF/LRVRF motif (Cohen, 2002), and only one GTS of CgPPP1C was found (data not shown). These results may indicate that GTS is not specific in oysters, suggesting the important role of CgPPP1R3B in glycogen metabolic regulation.

CgPPP1R3B interacts with the key glycogen metabolic enzymes CgGS and CgGP. CgPPP1R3B was shown to interact with both of these enzymes in this study. PTG could bind to the GS protein, and the binding site was shown to be in the C-terminus in mammalian cells (Printen et al., 1997; Fong et al., 2000). For PPP1R3B, researchers observed neither an interaction nor a binding site with GS (Armstrong et al., 1998; Newgard et al., 2000). In this study, we confirmed the interaction but did not find the binding site, which may indicate that the whole protein is needed for GS binding, and perhaps the misfolding of the truncated protein impaired the binding action (Fong et al., 2000). Moreover, GP interacts with CgPPP1R3B, but the interaction is not very strong, as shown by the Y2H assay. Initial studies reported that the GP binding site lies in the C-terminal 16 amino acids of PPP1R3B (*G*_L_) (Armstrong et al., 1998). This finding was supported by the observation that the C-terminal 16 amino acids of CgPPP1R3B showed very low similarity to those of the human, mouse and zebrafish orthologs (Supplementary Figure S3). Furthermore, CgPPP1R3B could interact with the oyster glycogen molecule by co-sedimentation assays *in vitro*. The CBM21 conserved domain in the middle of the protein is thought to be responsible for binding. This domain shared high similarity with those from different species (Printen et al., 1997; Armstrong et al., 1998; Fong et al., 2000).

Overexpression of *G*_M_, PTG, and PPP1R3B in cell lines and intact cells all increased the corresponding enzyme activity and glycogen levels. Overexpression of PTG in the liver of normal rats demonstrated that glycogen levels in fasted or fed PTG-overexpressing animals were 70% higher than those in fed controls (O’Doherty et al., 2000). Stender et al. (2018) found that overexpression of human PPP1R3B in HEK293 cells increased the glycogen level almost 20-fold. Because of the lack of mature mollusk cell lines, we overexpressed the CgPPP1R3B protein in HEK293 cells, and nearly twofold higher glycogen levels were observed in overexpression cells than in control cells. Due to the high conservation of PP1C protein, PP1C binding and glycogen binding sites, we concluded that CgPPP1R3B functioned well in HEK293 cells and thus accelerated glycogen accumulation. Transgenic mice with liver-specific deletion of PPP1R3B showed significantly reduced glycogen synthase protein levels and substantially decreased total hepatic glycogen content (Mehta et al., 2017). We found that RNAi of CgPPP1R3B significantly reduced the glycogen content compared with that of the control (*p* < 0.05). Given that glycogen metabolism in oyster showed seasonal variation (Bacca et al., 2005), we hypothesized that the regulation of oyster glycogen metabolism has a long-term regulation cycle compared to the quick fluctuation period for mammals (Stender et al., 2018), which may also explain why the decrease in glycogen level by RNAi was not as strong as that by transgenic deletion of the gene in mouse or human cells.

The subcellular locations of CgPPP1R3B, CgGS, CgPPP1C, and CgGP may imply that they participate in glycogen metabolism in the cytoplasm. Binding of GTS to glycogen metabolic enzymes may occur in the cytosol, followed by translocation of the formed complex to the glycogen particle (Newgard et al., 2000). Likely because of a redistribution of PPP1C and GS to glycogen particles, overexpression of PPP1R3B in cells strongly improves glycogen levels (Mehta et al., 2017).

Thus, CgPPP1R3B could interact with CgPPP1C, CgGS, and CgGP and target the glycogen molecule, forming a complex (Newgard et al., 2000), and CgPPP1R3B served as a molecular scaffold to connect the corresponding proteins. Moreover, PPP1C cannot interact with CgGS and CgGP directly (data not shown), which highlights the importance of PPP1R3B in this metabolic regulatory mechanism (Figure 5). CgPPP1C may activate CgGS and inactivate CgGP, thereby improving glycogen levels.

### Association of SNPs With Glycogen Content

Compared to growth traits, which have been relatively well studied and for which molecular breeding is practical (Gutierrez et al., 2018b), nutritional quality traits have rarely been used for genetic modification (Hollenbeck and Johnston, 2018). To date, less than ten glycogen associated SNPs have been identified in oysters. Candidate gene association studies showed that six SNPs in the coding region of CgGS were significantly associated with glycogen content (Liu et al., 2017) as well as two SNPs in Cg_GD1 (glycogen debranching enzyme) and one SNP in Cg-GP (glycogen phosphorylase) (She et al., 2015). A high-density genetic map found a QTL explaining 8.6% of glycogen phenotypic variation, and a new gene annotated as a zinc finger protein may participate in glycogen metabolism (Li C. et al., 2018). Many phenotypic variations remain to be explained.

In this study, 10 of 13 SNPs were confirmed to be associated with glycogen content in a wild population, eight of which were also associated with glycogen content in the GWAS population (Table 1). SNPs from this study could account for 1.4–6.3% of the PVE, which is comparable to the GWAS results for host resistance to *Ostreid herpesvirus* of oyster, with the top 10 markers ranging between 1.9 and 4.7% (Gutierrez et al., 2018a) as well as GWAS result of growth traits in juvenile farmed Atlantic salmon, with the top SNP explain ∼7% of the additive genetic variation. However, the use of selected fast growing lines may contribute to the high phenotypic variation of 7–52% PVE of growth traits in common carp (Su et al., 2018). Moreover, minor alleles were the favorable allele for 8 of the 10 SNPs, indicating that we should pay more attention to the minor alleles. Zhou et al. (2018) found that the MAF of the most significant SNP (carp159317) was significantly different between individuals with scattered scale patterns and individuals with normal scale patterns in the Yellow River carp (0.49 and 0, respectively). Holborn et al. (2018) reported one favorable allele was minor allele (MAF = 0.078) for bacterial kidney disease resistance in North American Atlantic salmon population.

Associated SNPs were not in the same LD block, and the combination of favorable genotypes showed potential for selective breeding for high glycogen content using these markers. On the one hand, we can genotype the oysters with these markers before spawning and select individuals with favorable combinations as parents, which may increase the glycogen content by approximately 14.2% on average. On the other hand, individuals with favorable genotypic combinations comprised 4.9% of the wild population, indicating it is feasible to conduct molecular breeding to improve glycogen content using this favorable genotypic combination.

### Regulatory Mechanism of SNP-Mediated Glycogen Content

These associated SNPs were distributed in different regions of the CgPPP1R3B gene, which suggested different mechanisms of glycogen content regulation. Among the 10 significant SNPs, three SNPs were located in the 5′ flanking region spanning 100–500 bp upstream of transcription start sites (TSS). These SNPs are located in the promoter region and may influence the transcription process. This hypothesis was confirmed by the fact that different genotypes of the SNPs 63644, 63965, and 63878 showed different expression levels. To date, few reports have focused on oyster gene promoter regions and functions. In other invertebrates, such as fruit flies, most of the promoters lie in the 1-kb region, and some of them may affect the TSS (Schor et al., 2017). This may have a distinct regulatory effect of different genotypes of the genetic variants, confirming the *in vivo* expression differences (Cannavò et al., 2017). Moreover, one SNP among the 10 associated SNPs located in the 5′UTR and the expression level of CgPPP1R3B presented differences among different genotypes. Genetic variations in the 5′UTR may impact RNA stability and translation efficiency, as confirmed in humans and plants (Zou et al., 2003; Dahlqvist et al., 2010). Furthermore, one associated synonymous SNP (78604) was located in the CDS. Two synonymous SNP in Serum amyloid A gene were associated with Vibrio-resistance in the clam Meretrix meretrix (Zou and Liu, 2015). Komar (2007) reported that naturally occurring synonymous SNPs can affect *in vivo* protein folding and combinations of three SNPs for a gene that altered glycoprotein activity. Finally, five SNPs were located in introns, a intronic SNP was found to be associated with resistance to sea lice in Atlantic salmon (Correa et al., 2017) and two intronic SNP in Serum amyloid A gene in the clam M. meretrix were significantly associated with Vibrio-resistance and one intronic SNP was associated with growth related traits by candidate gene association studies (Zou and Liu, 2015). A 311-bp deletion in intron 10 and exon 11 of *fgfr1A* was proved to be the causal gene responsible for abnormal scattered scale in the Yellow River carp (Zhou et al., 2018). Several intronic SNPs were confirmed to be associated with glucose metabolism in humans (Bouatia-Naji et al., 2008). Intronic SNPs may impact mRNA processing, the precise mechanism requires further elucidation in the future.

## Conclusion

The CgPPP1R3B protein can bind CgPPP1C, CgGS, CgGP and glycogen molecules, thus participating in glycogen metabolic regulation. Associated SNPs may increase the transcription level of CgPPP1R3B, thereby increasing the glycogen content of oyster individuals. These associated SNPs or favorable genotypic combinations provide potential markers for marker-assisted selection programs for high-glycogen oyster breeding.

## Author Contributions

LL and GZ conceived and designed the study. SL and WW collected the experimental materials. SL and BH contributed to the experimental work. SL, JM, and KS performed the most of the statistical analyses. SL and LL wrote and polished the manuscript. All authors read and approved the final manuscript.

## Conflict of Interest Statement

The authors declare that the research was conducted in the absence of any commercial or financial relationships that could be construed as a potential conflict of interest.

## Footnotes

1 http://www.ncbi.nlm.nih.gov/gorf/orfig.cgi

2 https://www.uniprot.org

3 http://www.ncbi.nlm.nih.gov/guide/proteins/
