# Supplementary material for: Association and Functional Analyses Revealed That PPP1R3B Plays an Important Role in the Regulation of Glycogen Content in the Pacific Oyster Crassostrea gigas
Source: Front Genet. 2019 Feb 14;10:106. doi: 10.3389/fgene.2019.00106 (PMC6396720; doi:10.3389/fgene.2019.00106)
Supplement: Supplementary file 2 [file Data_Sheet_2.docx]

Supplementary Material

Association and functional analyses revealed that PPP1R3B plays an important role in the regulation of glycogen content in the Pacific oyster *Crassostrea gigas*

Sheng Liu^1, 2, 3^, Li Li*^1, 4, 5, 6^, Jie Meng^1, 4, 5, 6^,

Kai Song^1, 4, 5, 6^, Baoyu Huang^1, 2, 5, 6^, Wei Wang^1, 2, 5, 6^, Guofan Zhang*^1, 2, 5, 6^

Affiliations and addresses:

^1^ Key Laboratory of Experimental Marine Biology, Institute of Oceanology, Chinese Academy of Sciences, Qingdao 266071, China

^2^ Laboratory for Marine Biology and Biotechnology, Qingdao National Laboratory for Marine Science and Technology, Qingdao 266071, China

^3^ University of Chinese Academy of Sciences, Beijing 100039, China

^4^ Laboratory for Marine Fisheries Science and Food Production Processes, Qingdao National

Laboratory for Marine Science and Technology, Qingdao 266071, China

^5^ National and Local Joint Engineering Laboratory of Ecological Mariculture, Qingdao 266071 China

^6^ Center for Ocean Mega-Science, Chinese Academy of Sciences, Qingdao 266071, China

***Correspondence**: Li Li, [lili@qdio.ac.cn](mailto:lili@qdio.ac.cn). Guofan Zhang, [gzhang@qdio.ac.cn](mailto:gzhang@qdio.ac.cn)

## Supplementary Figures


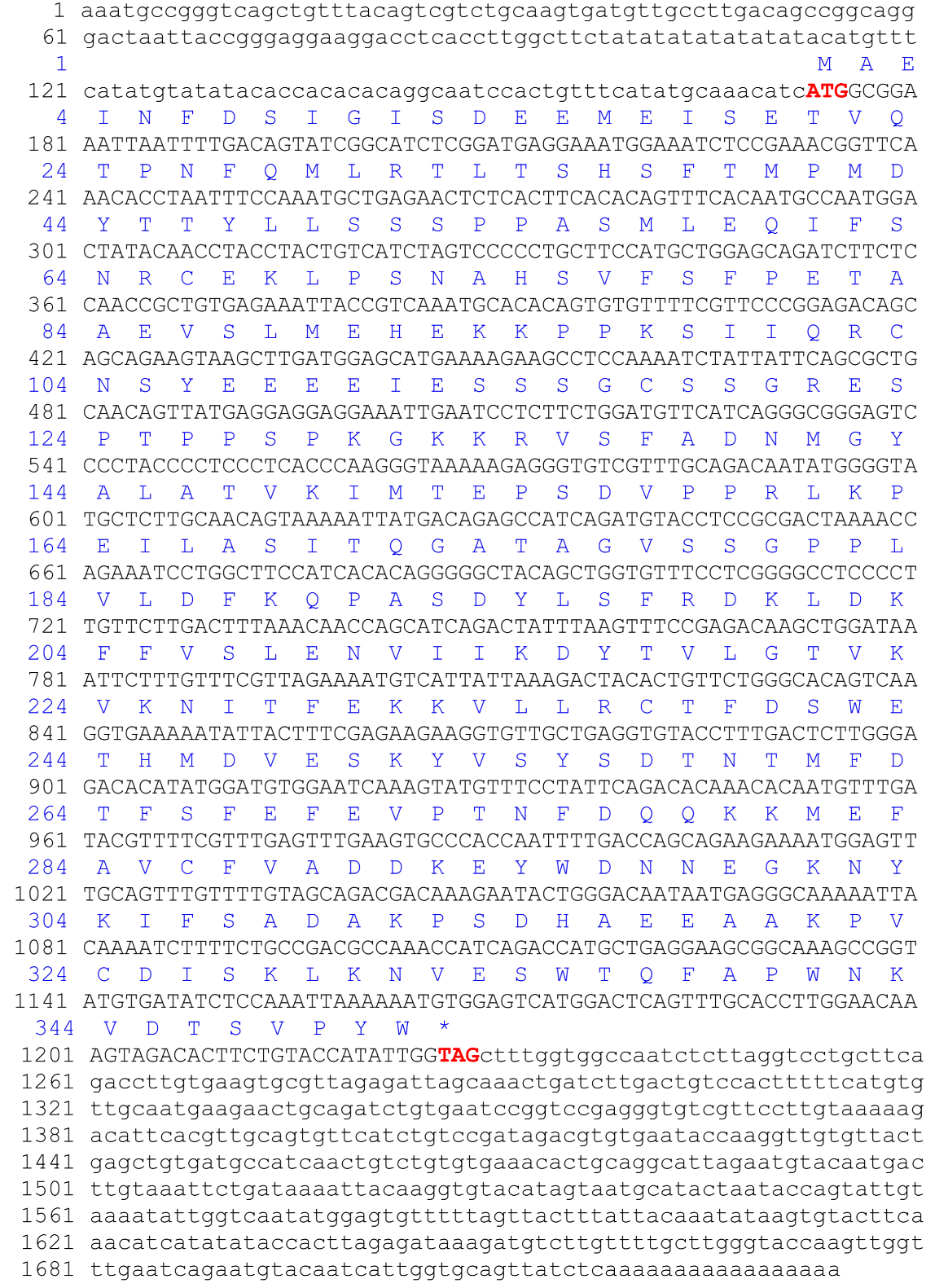


Supplementary Figure 1. CgPPP1R3B cDNA sequence and amino acid encoded. The capital letters are protein encoded sequences, the start codon (ATG) and stop codon (TAG) are in red bold.

The lowercase letters are un-translation region.


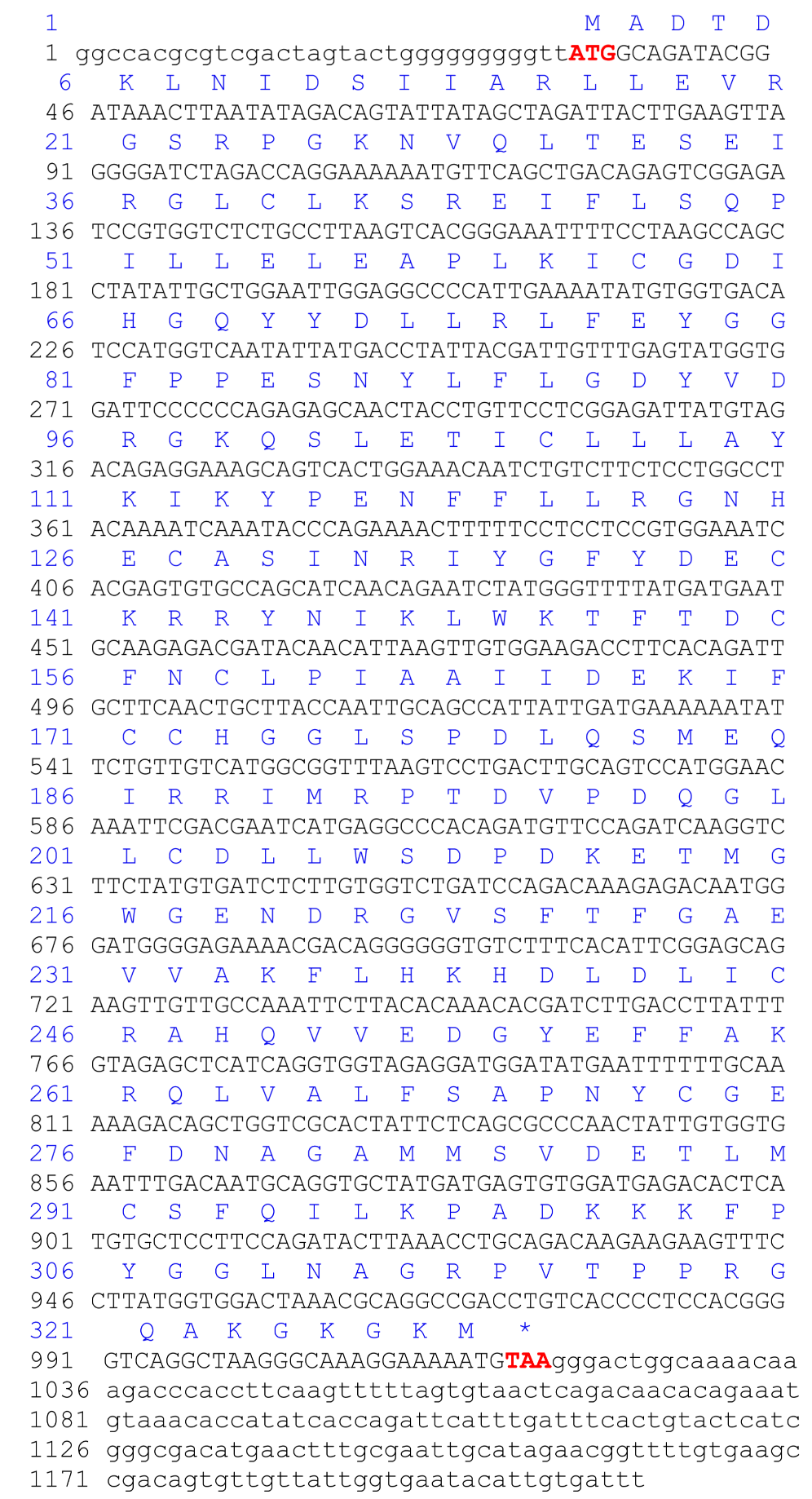


Supplementary Figure 2-CgPPP1C cDNA sequence and amino acid encoded. The capital letters are protein encoded sequences, the start codon (ATG) and stop codon (TAA) are in red bold.

The lowercase letters are un-translation region.


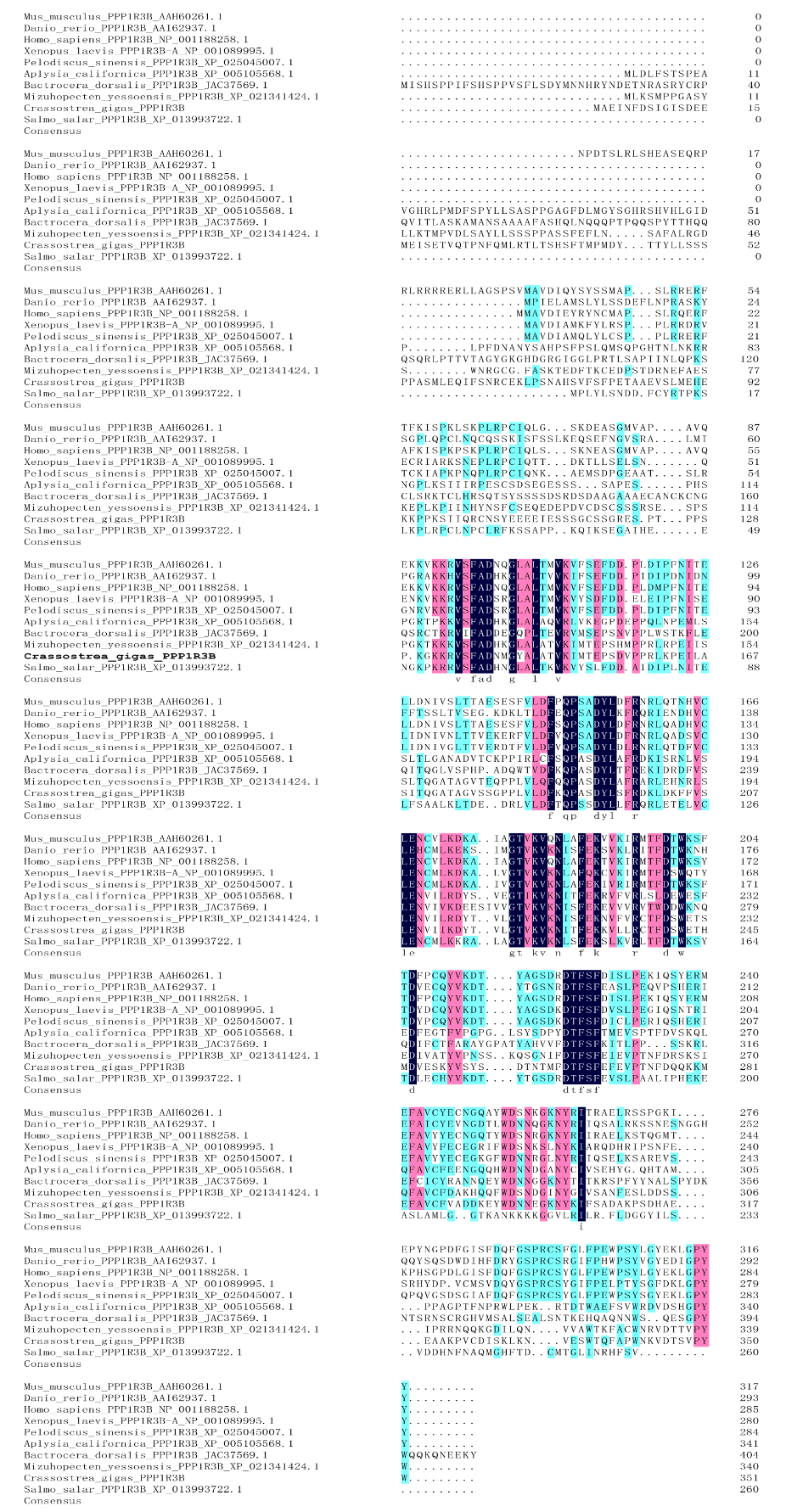


Supplementary Figure 3-Multiple alignment of PPP1R3B protein sequence of human, mouse, amphibian, reptile, fishes, oyster and other shellfish, protein sequence were downloaded from NCBI (the serial number after the Latin name of the species).


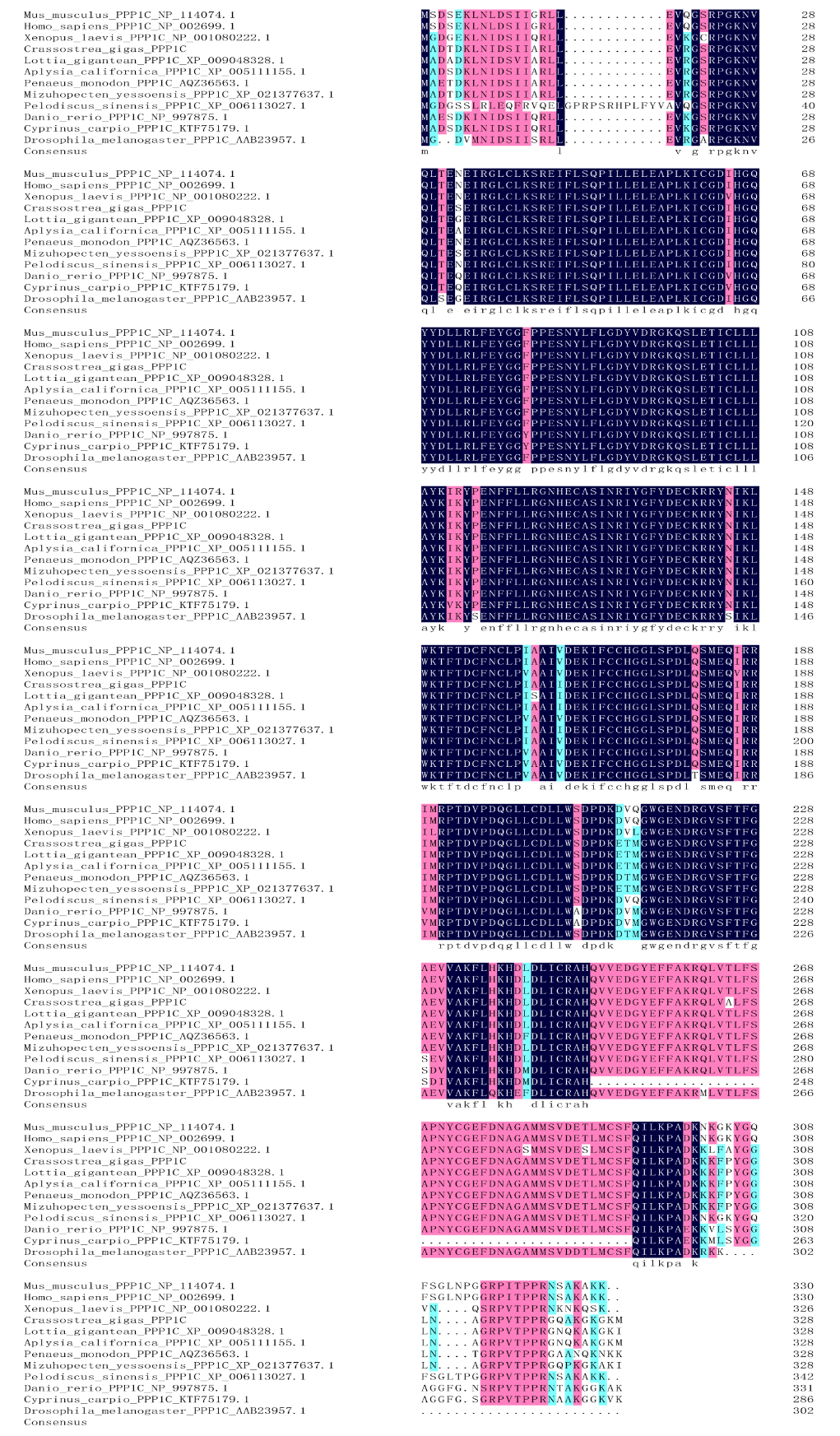


Supplementary Figure 4: Multiple alignment of PPP1C protein sequence of human, mouse, amphibian, reptile, fishes, oyster and other shellfish, protein sequence were downloaded from NCBI (the serial number after the Latin name of the species).


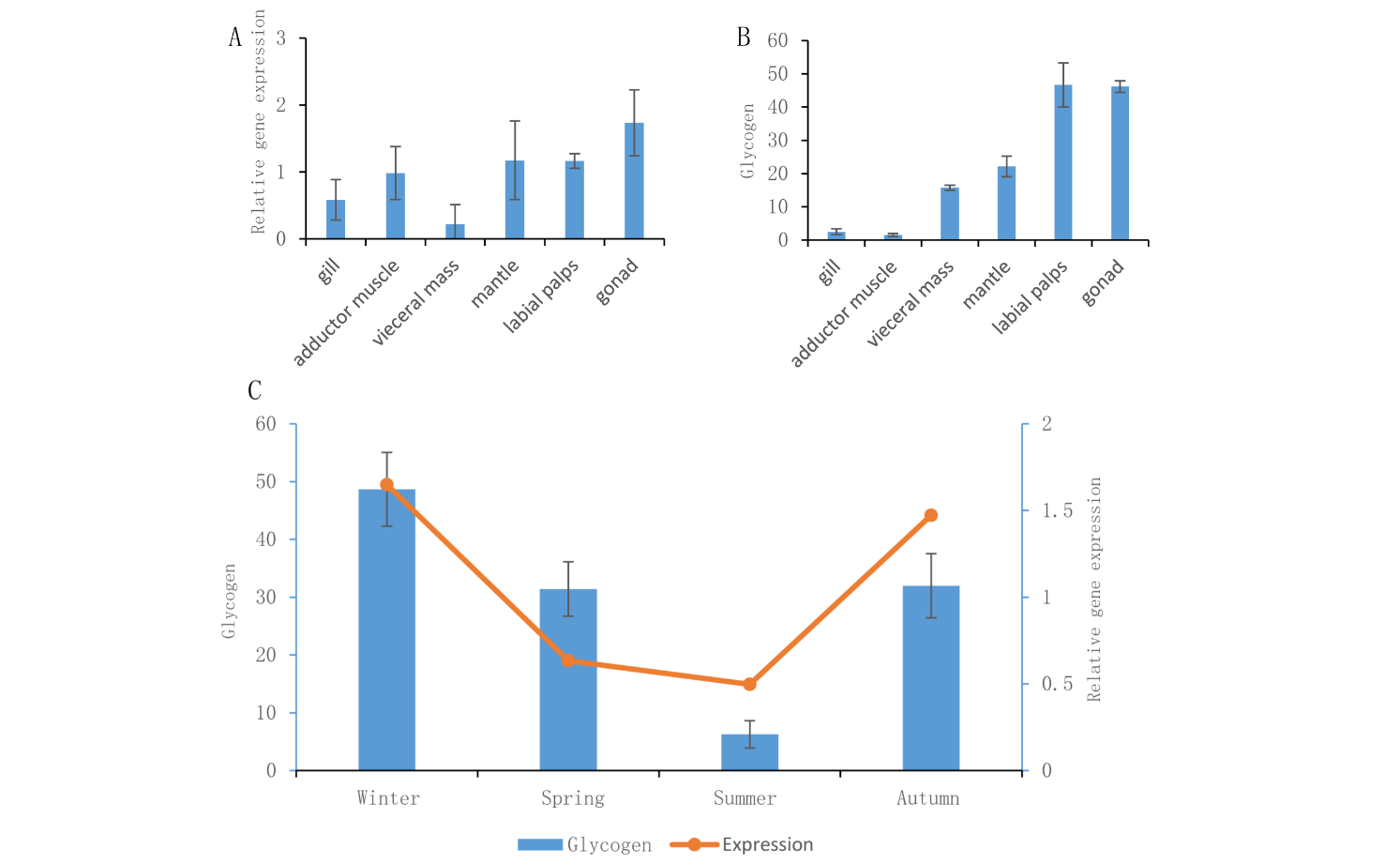


Supplementary Figure 5: Gene expression profile of CgPPP1R3B in (A) different tissues, (B) glycogen content in different tissues, and (C) different season around year round. Vertical bars represent the mean ±SEM (n = 15).


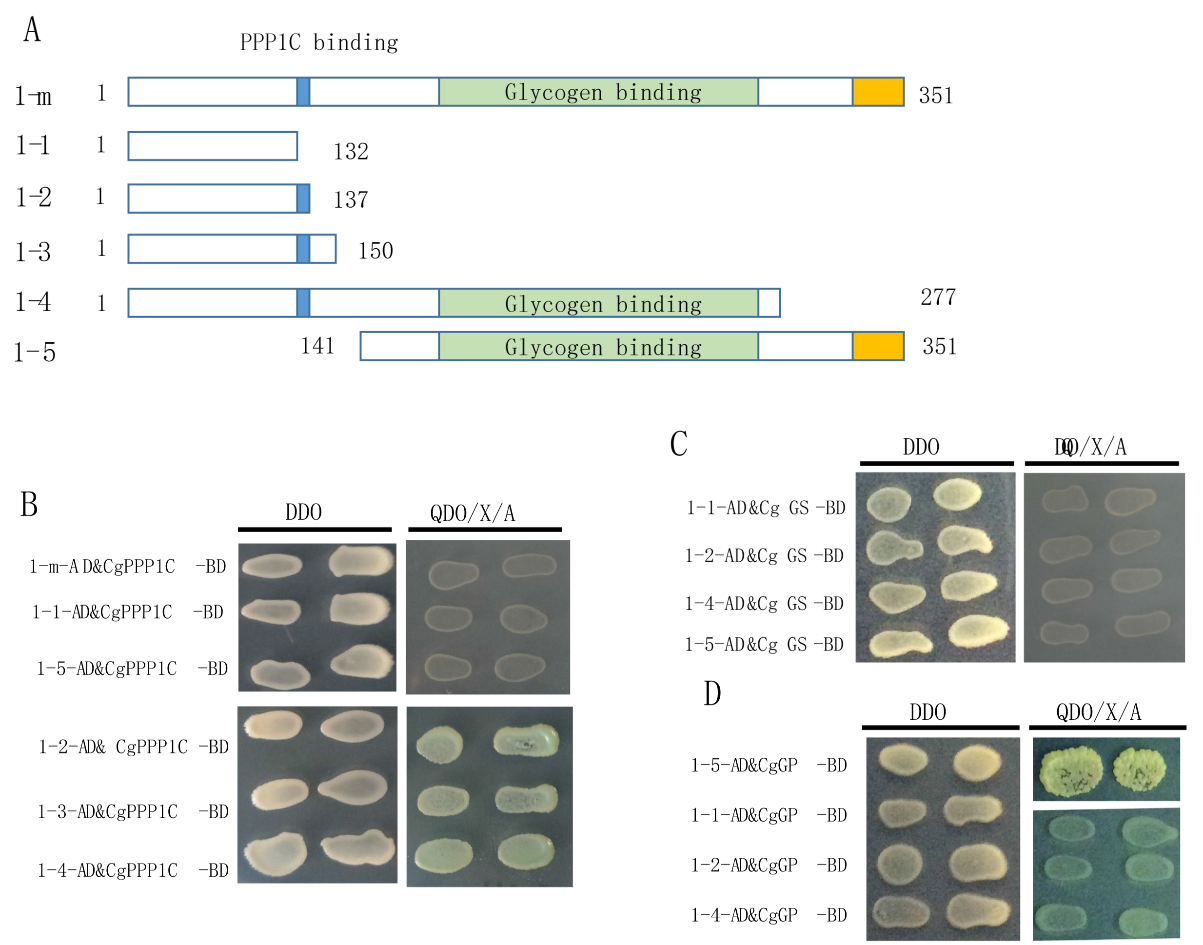


**Supplementary Figure 6.** Active domain of CgPPP1R3B interact with CgPPP1C, CgGS and CgGP.

(A): Truncated protein of CgPPP1R3B constructed, 1-m means the site-directed mutagenesis of V135E, and 1-1 to 1-6 represent other truncated protein with the sequence behind. (B) Active motif CgPPP1R3B interact with CgPPP1C tested by Y2H, controls were not shown. (C) Active domain CgPPP1R3B interact with CgGS by Y2H, experiment assay were shown, without showing control. (D) Active domain of CgPPP1R3B interact with CgGP verified by Y2H. DDO: -Leu/-Trp double-dropout media, QDO/X/A:-Ade/-His/-Leu/-Trp quadruple-dropout media with X-α-Gal and Aureobasi A.


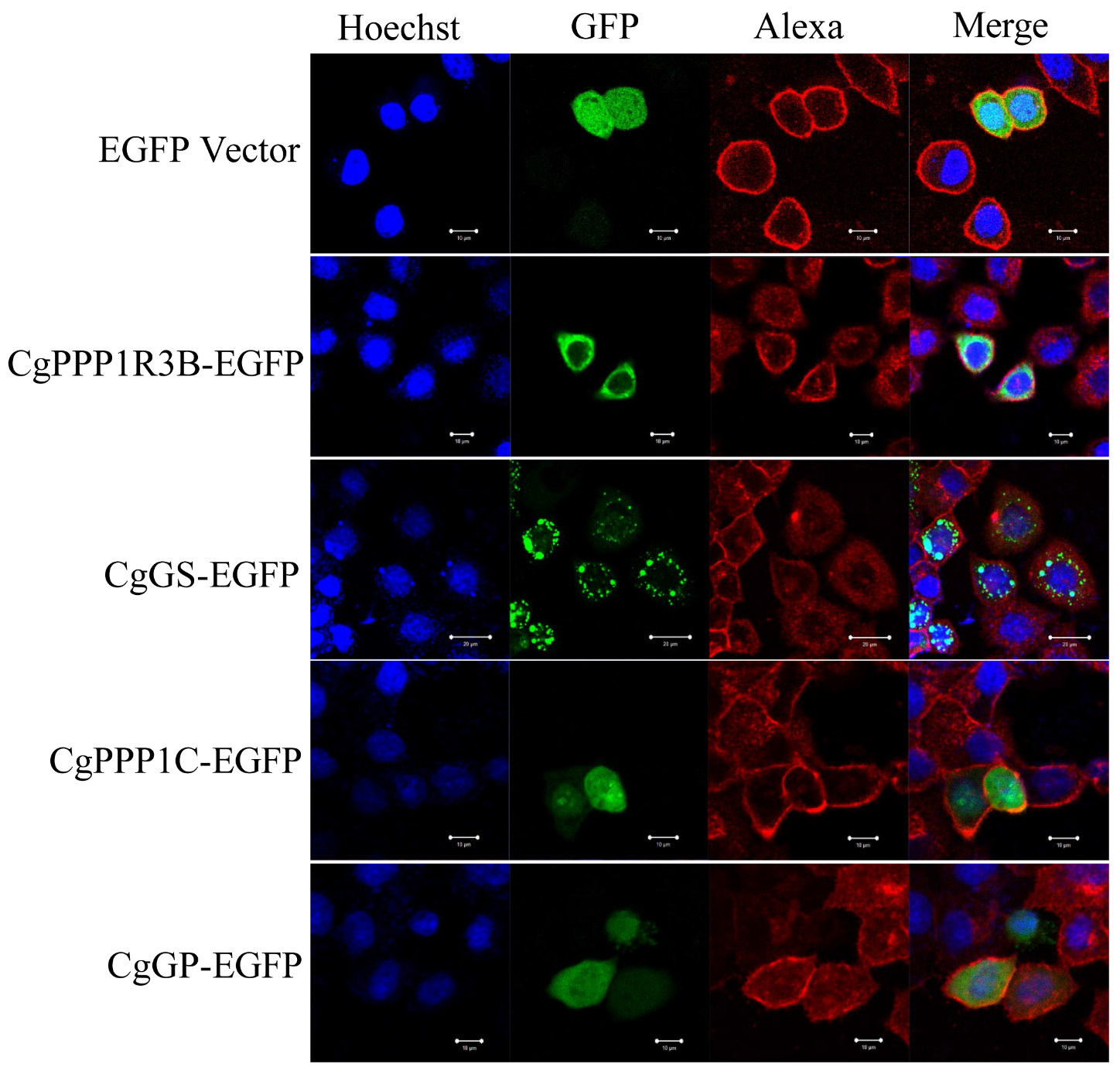


Supplementary Figure.7 Subcellular location of EGFP Vector (pEGFP-N1), CgPPP1R3B, CgGS, CgPPP1C and CgGP in HeLa cells. Nuclei was counterstained with Hoechst 33342 (blue), membrane was counterstained with Alexa Fluor 594(red). The upper panels depict localization of the EGFP negative control, and the lower four panels showed localization of the CgPPP1R3B, CgGS, CgPPP1C and CgGP protein.


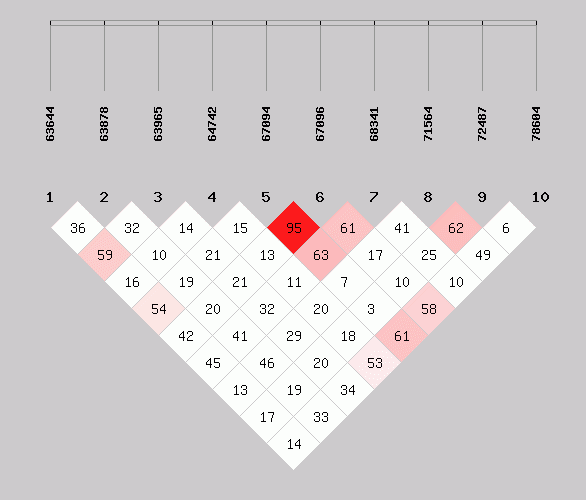


Supplementary Figure 8: Pairwise linkage disequilibrium (LD) D' graph for the ten SNPs of the CgPPP1R3B gene, ID of SNPs were showed in the upper. D'>80 was thought to be the threshold of linkage or not. The darker of the color, the much linked of the SNPs.
